# Assessment of Antioxidant and Antimutagenic Properties of Red and White Wine Extracts In Vitro

**DOI:** 10.3390/metabo11070436

**Published:** 2021-07-02

**Authors:** Fotios Tekos, Sotiria Makri, Zoi-Vasiliki Skaperda, Anastasia Patouna, Kallirroi Terizi, Ioannis D. Kyriazis, Yorgos Kotseridis, Eleni Vaskani Mikropoulou, Georgios Papaefstathiou, Maria Halabalaki, Kouretas Demetrios

**Affiliations:** 1 Department of Biochemistry and Biotechnology, University of Thessaly, Viopolis, Mezourlo, 41500 Larissa, Greece; ftekos@uth.gr (F.T.); sotirina_m@hotmail.com (S.M.); zoskaper@bio.uth.gr (Z.-V.S.); anastasia.pat93@hotmail.com (A.P.); kterizi@outlook.com (K.T.); ioankyriazis@uth.gr (I.D.K.); 2 Laboratory of Oenology, Department of Food Science & Human Nutrition, Agricultural University of Athens, 75 Iera Odos, 11855 Athens, Greece; ykotseridis@aua.gr; 3 Department of Pharmacy, Division of Pharmacognosy and Natural Products Chemistry, National and Kapodistrian University of Athens, Panepistimioupoli Zografou, 15771 Athens, Greece; E.Mikropoulou@pharm.uoa.gr (E.V.M.); gpapaefstath@pharm.uoa.gr (G.P.); mariahal@pharm.uoa.gr (M.H.)

**Keywords:** wine, antioxidants, polyphenols, free radicals, oxidative stress

## Abstract

Wine is an alcoholic beverage of complex composition obtained through the fermentation of grape must. The consumption of wine has already been associated with a multitude of beneficial effects due to its high polyphenolic content. In this study, four Greek emblematic wines from two red (i.e., Xinomavro and Agiorgitiko) and two white (i.e., Assyrtiko and Malagouzia) varieties were analyzed for the estimation of their antioxidant profiles. To address this question, we assessed their ability to scavenge both synthetic and endogenous free radicals, such as DPPH^•^, ABTS^+^^•^, OH^•^, O_2_^−^, their potential reducing power, and their antimutagenic and antigenotoxic properties. All varieties exhibited potent antioxidant activity, as indicated by the results of methods above, with the red wines appearing more effective than the white ones regarding antioxidant capacity. Our small-scale study is the first to reveal that these wine varieties may have the ability to scavenge the most reactive endogenous radicals. In the future, this finding must be accompanied by larger studies to fill a knowledge gap in the scientific literature concerning a holistic approach of the in vitro antioxidant action of plant polyphenolic compounds. Conclusively, we believe that wines possess high bioactivity that allow them to settle in the industry of food additives and medicinal products.

## 1. Introduction

The term “Oxidative stress” was firstly described by H. Sies (1985) as “the imbalance between oxidants and antioxidants in favor of the oxidants, potentially leading to damage”, and was redefined from D. Jones (2006) as “a disruption of redox signaling and control.” [1,2]. Oxidative stress can occurs in a system when the production of free radicals exceeds its antioxidant defense mechanisms [3]. Free radicals can cause detrimental effects in crucial cellular biomolecules, such as proteins, lipids, and DNA, and can eventually promote or contribute to a number of diseases, including cancer, cardiovascular complications, diabetes, rheumatoid arthritis, Parkinson’s disease, Alzheimer’s disease, and Huntington’s disease [4,5,6,7,8].

Antioxidants from natural sources have already been used to enhance the antioxidant defense system by preventing the harmful effects of aberrant oxidative stress. Natural products and foods constitute the main sources of antioxidants. Scientists have attempted to define the possibility of these products to have bioactive compounds that possess the required ability to counteract oxidative stress, thus alleviating chronic diseases manifestations [9,10,11,12]. The most studied chemical compounds, widely known for their biological activities, are polyphenols, the main bioactive phytochemicals in foods characterized by the presence of cyclic benzene compounds [10,13]. More than 8000 polyphenols have been recognized in plants, including at least 4000 flavonoids [13]. These compounds are mostly found in fruits, cereals, vegetables, and beverages [14,15]. Phenolic acids, flavonoids, stilbenes, and lignans are among the main polyphenolic categories that exert antioxidant activity by scavenging free radicals and disrupting oxidative reactions [14,16]. Studies with diets rich in these polyphenolic compounds have been found to shield from oxidative stress and protect from a wide range of diseases such as osteoporosis, cancer, diabetes, cardiovascular, and neurodegenerative diseases [6,17,18,19,20,21]; these studies add a translational value to the in vitro studies that have investigated the antioxidant potential of new compounds from natural products or foods.

Between 1958 and 1964, Ancel Keys proposed the concept of a different diet model, named the “Mediterranean Diet”, through his “Study of the Seven Countries” [22]. In this study, different dietary habits from cohorts in the USA, Japan, Finland, the Netherlands, former Yugoslavia, Italy, and Greece were compared, and the diet ingredients that were associated with a higher life expectancy and a lower occurrence of chronic diseases were defined [22]. More elaborately, this study analyzed the impact of a high intake of antioxidants through the consumption of wines, apart from the common consumption of fruits, vegetables, and olives [23]. Due to the fact that the Mediterranean Diet is based on a great variety of food and focuses on consumption frequency, it was declared as an “Intangible Cultural Heritage of Humanity” in 2010 by UNESCO [24].

Wine, a characteristic alcoholic beverage consumed in the Mediterranean diet, has complex composition that is obtained through the fermentation of grape must. The quality and variety of grapes used in the vinification process have the highest impact on the composition of wine [25,26]. Thereafter, polyphenols’ diversity and levels are highly dependable due to different grape varieties (white or red) and their geographical origin [27]. Specifically, red wines contain more than 500 compounds, with water, alcohol (ethanol), and polyphenols (flavonoids and non-flavonoids) representing their major constituents [25,28,29]. It has already been reported that red wine has 10-fold more polyphenol enrichment compared to white wine; this is due to winemaking protocols [25,26]. This degree polyphenol enrichment constitutes the only major component difference between red and white wines. Among polyphenols, flavonoids such as flavones, flavan-3-ols, flavonols, anthocyanins, and tannins are plant-derived compounds with antioxidant properties [30]. They account for over 85% of the phenolic components in red wines, providing the characteristic red color and distinctive taste; they are also implicated in exerting beneficial properties on human health, including cardiovascular protection effects [25,31]. On the other hand, the contribution of non-flavonoids, such as stilbenes, benzoic acids, and cinnamic acids, to the potential bioactive properties of wine is still under scientific debate [31]. Nevertheless, resveratrol, the non-flavonoid compound mainly contained in red wines, as well as its derivatives, such as glucoside [32] and oligomers [33,34], exert a significant chemoprotective activity via their antioxidant, anti-inflammatory and cytoprotective properties [29,35,36,37]. Zurine et al. highlight the effectiveness of the bioactive phenolic compounds contained in grapes and wines that impart antioxidant, cardioprotective, anti-cancer, anti-inflammatory, anti-aging, and antimicrobial properties, and also alleviate manifestations of type 2 diabetes [38]. At the molecular level, the antioxidant properties of resveratrol and polyphenols present in wine have been associated with concomitant increases in antioxidant enzymes and decreases in reactive oxygen species generation [36,39,40]. At the clinical level, according to a concept observed in France in the 1980s known as “The French Paradox”, moderate consumption of alcoholic beverages, especially wine, was able to provide protection from cardiovascular diseases [31,41,42]. Although this paradox has been criticized [43,44], epidemiological studies on Mediterranean populations, have shown a lower incidence of coronary heart disease (CHD), though this has been attributed to the consumption of antioxidant-rich foods [45,46]. Other studies have associated the beneficial properties of wine consumption with the occurrence of chronic neurodegenerative diseases (Alzheimer’s and Parkinson’s disease), diabetes, aging, as well as strengthening of the immune system via vitamins K, A, and C [47,48].

A plethora of studies have already been published for the purpose of determining the antioxidant capacity of wines. In a previous study from our lab, a holistic approach concerning the assessment of the antioxidant action of plant-derived polyphenolic compounds was developed [49,50]. In this regard, it is indispensable to evaluate the potential antioxidant, antimutagenic, and DNA protective activities of four Greek wine varieties, two red and two white, using a combination of customary tools. The aim of the present study is to screen and estimate the antioxidant capacity of four emblematic Greek wine varieties: Xinomavro, Agiorgitiko, Assyrtiko, and Malagouzia. For that reason, four extracts of these wine varieties were tested for their ability to scavenge synthetic radicals (DPPH^•^, ABTS^•+^) and endogenous radicals of ^•^OH and ^•^O_2_^−^. Moreover, we determined the ability of these extracts to reduce Fe^3+^ to Fe^2+^ through their reducing power and as well, the protective potential of wine extracts against the mutagenic effects of DNA strand breaks that are due to induced peroxyl radical generation and tert-induced mutagenicity, respectively. The aforementioned readouts comprise only the introductory level for determining the antioxidant capacity of these wine varieties and any plant-derived extract.

## 2. Results

### 2.1. UHPLC-ESI-TripleTOF-HRMS Analysis and Metabolites Comparison

**Figure 1:**
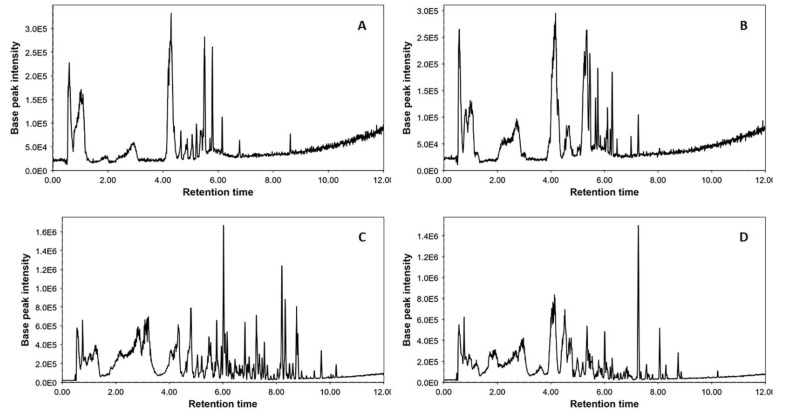
LC-ESI(-)-HRMS profiles of Greek wine samples: (**A**) Assyrtiko, (**B**) Malagouzia, (**C**) Agiortgitiko, and (**D**) Xinomavro.

In the present study, LC-HRMS/MS profiling methods were used for the identification of wine constituents, mainly phenolics, with an interest in phenolic acids and flavonoids. For this purpose, the suspected screening streamline was employed for identification purposes and for the monitoring of secondary metabolite levels that have previously been reported in wine samples. Briefly, all wine samples under analysis were screened for the presence of 65 compounds already reported [51,52,53] by incorporating the Sciex OS Analytics platform (Figure 1). Tentative identification was based on the “Formula finder” score, the isotopic ratio match, and on the HRMS/MS spectra. After eliminating features that were detectable at trace levels, or not present in all four varieties, 29 compounds were prioritized and their relative concentration levels were assessed and compared (Table 1 and Table S1).

**Figure 2:**
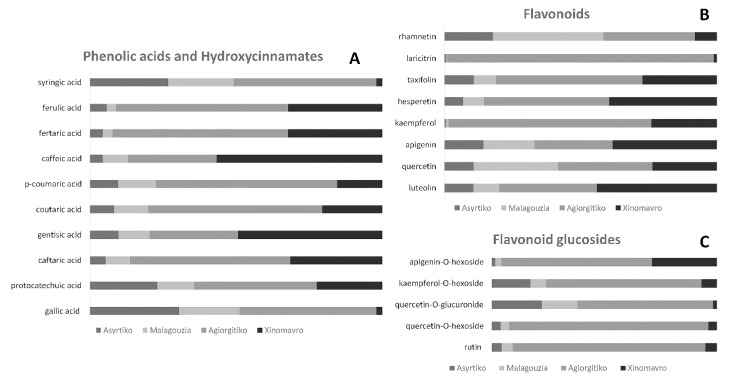
Comparative representation of (**A**) phenolic acids and hydroxycinnamates, (**B**) flavonoid, and (**C**) flavonoid glucoside in all wine extracts.

Based on the profiling results, all analyzed extracts demonstrated a rich phytochemical profile (Figure 1), with red wine extracts being superior compared to white wine extracts in detected features and metabolite variability. Most detected compounds belonged to the chemical classes of flavonoids, phenolic acids, and small phenols such as tyrosol and hydroxytyrosol. Moreover, the hydroxycinnamates caftaric and coutaric acids were both abundantly present in all wine samples. Overall, white wine varieties exhibited poorer detectable features in their chemical profiles; however, in the few minor metabolites that were detected in traces, such as syringic and ellagic acid, levels were even higher compared to Agiorgitiko or Xinomavro wine samples (Table S1, Figure 2). To our surprise, resveratrol was only detected at low levels in the Malagouzia variety extract. The inter-sample comparative representation of the distinct phenolic compound families is shown in Figure 2.

### 2.2. In Vitro Measurements for the Assessment of the Wine Extracts’ Antioxidant Activity

#### 2.2.1. Total Phenolic Content of Wine Varieties (TPC)

Total Phenolic Content (TPC) was determined in all four Greek wine extracts. Analysis revealed that the red wine varieties, Agiorgitiko and Xinomavro, demonstrated higher phenolic content compared to the extracts derived from the white wines, Assyrtiko and Malagouzia (Table 2).

#### 2.2.2. Determination of IC_50_ Values of Extracts in DPPH^•^, ABTS^•+^, Reducing Power, Superoxide, and Hydroxyl Radical Scavenging Activity Assays

**Figure 3:**
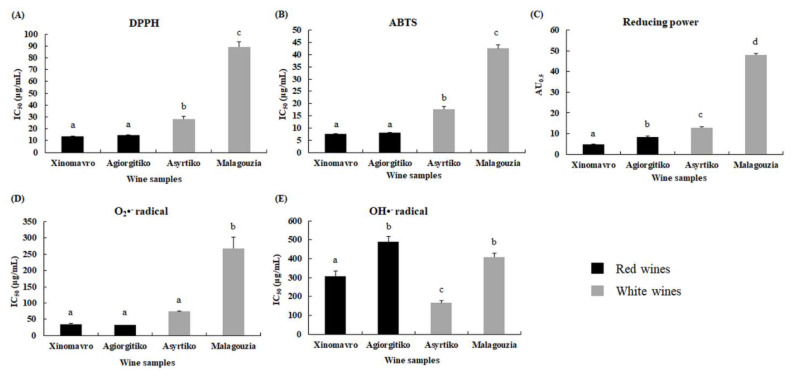
Antioxidant capacity of Greek wine extracts in in vitro assays. Results are expressed as mean ± SD. a–d: means without a common letter depict significant difference (*p* < 0.05).

All extracts from our wine samples exhibited strong antioxidant activity. More specifically, according to the DPPH^•^ assay, the IC_50_ values of the Xinomavro-, Agiorgitiko-, Assyrtiko-, and Malagouzia-derived extracts were 13.4, 14.5, 28.4, and 89.4 μg/mL, respectively (Figure 3A). Statistical analysis revealed that Xinomavro exerted stronger antioxidant activity compared to Assyrtiko (*p* = 0.012) and Malagouzia (*p* < 0.001), but this was not the case in Agiorgitiko (*p* = 0.969). Moreover, the extract from Agiorgitiko was the second most active compared to Assyrtiko (*p* = 0.016) and Malagouzia (*p* < 0.001). Finally, Assyrtiko-derived extract had the highest antioxidant activity in the DPPH^•^ assay among the white wine extracts (*p* < 0.001). Similar results were obtained with the ABTS^•+^ assay. IC_50_ values were determined at 7.3, 8.2, 18.4, and 43.5 μg/mL for Xinomavro-, Agiorgitiko-, Assyrtiko-, and Malagouzia-derived extracts, respectively (Figure 3B). Specifically, there was no significant difference between the Xinomavro- and Agiorgitiko-derived extracts (*p* = 0.720), but the Xinomavro-derived extract had a higher antioxidant capacity than Assyrtiko- (*p* = 0.001) and Malagouzia-derived extracts (*p* < 0.001). Additionally, no difference was observed between the Assyrtiko- and Malagouzia-derived extracts (*p* < 0.001) (Figure 3B).

As assessed by a reducing power assay, all wine extracts differed significantly from one other, with AU_0.5_ values of the Xinomavro-, Agiorgitiko-, Assyrtiko-, and Malagouzia-derived extracts being determined at 4.9, 8.3, 13.0, and 48.1 μg/mL, respectively (all *p* values < 0.01; Figure 3C).

Subsequently, the Malagouzia-derived extract had the lowest superoxide radical scavenging ability among the extracts tested. Specifically, IC_50_ values of the Xinomavro-, Agiorgitiko-, Assyrtiko-, and Malagouzia-derived extracts were measured at 34.5, 32.0, 73.9, and 268.5 μg/mL, respectively. Statistical analysis revealed no difference among the Xinomavro-, Agiorgitiko-, and Assyrtiko-derived extracts (*p* values ranged from 0.203 to 0.999). On the contrary, all these wine extracts exhibited increased antioxidant activity compared to Malagouzia-derived extract (*p* = 0.001) (Figure 3D). Of note, even though the red wine-derived extracts showed stronger antioxidant activity than the white wine-derived extracts, a hydroxyl radical assay revealed that the Assyrtiko-derived extracts possessed the lowest IC_50_ value (165.7 μg/mL), while the respective IC_50_ values of the Xinomavro-, Agiorgitiko-, and Malagouzia-derived extracts were 304.8, 491.2, and 409.1 μg/mL (*p* values ranged from 0.001 to 0.015; Figure 3E). Among the remaining three extracts, the Xinomavro-derived extract was more potent than the Agiorgitiko- (*p* = 0.006) and Malagouzia-derived extracts (*p* = 0.042), while the Assyrtiko-derived extract detected no significant difference between the Agiorgitiko- and Malagouzia-derived extracts (*p* = 0.095).

#### 2.2.3. Antigenotoxic Activity of Wine Extracts via a Plasmid Relaxation Assay

A plasmid relaxation assay revealed that the Agiorgitiko-derived extract had a stronger antigenotoxic activity compared to the Xinomavro-derived extract (*p* = 0.006), while no significant difference was detected among extracts from white wine (*p* = 0.076). Table 3 showcases the IC_50_ and IC_20_ values of the four Greek wine extracts tested.

#### 2.2.4. Antimutagenic Capacity of Wine Extracts through an Ames Test

An Ames test performed to determine the possible antimutagenic capacity of the four Greek wine extracts. Results indicated a distinct difference in IC_50_ values among all extracts tested (Table 4). Although the extracts derived from the two white wines differed significantly, with their IC_50_ values determined at 16.9 and 25.9 μg/mL for Assyrtiko- and Malagouzia-derived extracts (*p <* 0.001), respectively, no significant difference was revealed between the antimutagenic activity of the two red wine extracts (*p* = 0.275). On the contrary, each red wine extract was more potent than its white wine counterpart, since the former’s IC_50_ values were lower than those of the white wines (*p* < 0.001 in all comparisons).

## 3. Discussion

Our study has shown that both red and white wine varieties contain compounds that exhibit potent antioxidant activity showing their ability to scavenge synthetic endogenous radicals such as hydroxyl radical and superoxide anion. Notably, red wine extracts appear to be more effective than the respective extracts derived from white varieties, which is due to the former’s higher polyphenolic content, as determined via a Folin-Ciocalteu assay.

**Figure 4:**
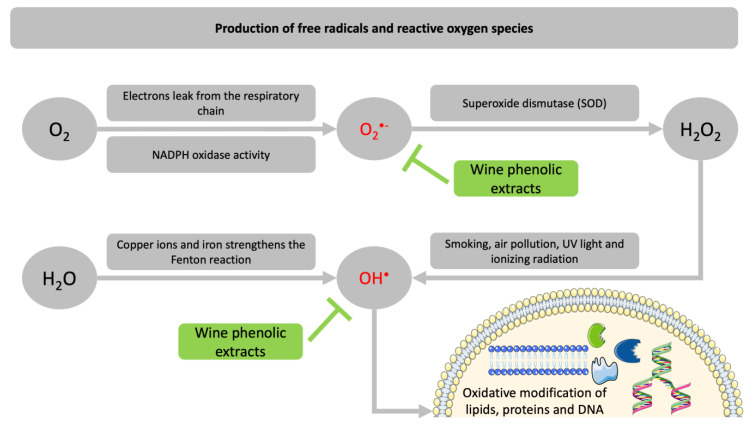
Production of free radicals and reactive oxygen species. O_2_^−^: superoxide radical, OH: hydroxyl radical, H_2_O_2_: hydrogen peroxide.

Oxidative stress has been implicated as a contributing factor in the pathogenesis of many clinical conditions [54] (Figure 4). Therefore, therapies that aim to strengthen antioxidant potential could alleviate several diseases. Although the French paradox has been criticized [43,44], it served as the commencement of what became the onset for many studies that examined the effects of wine consumption on human health, as well as the antioxidant and anticancer properties that their ingredients possess [55,56,57]. Even though grape stem extracts have different biochemical content compared to wines, they have also been used in experimental models for the designation of grape polyphenols’ capabilities regarding their protective role in relation to oxidative modifications. It has been reported in previous literature that grape stem extracts possess important bioactivities that are beneficial for human health; thus, they can be exploited as food additives for generating biofunctional foods with enhanced values [58].

The literature lacks a standardized array of methodologies for evaluating the biochemical properties of foods through a standard series of readouts in order to avoid variabilities from different experimental conditions and approaches [50]. Thus, we found it appropriate to initially evaluate the potential antioxidant, antimutagenic, and DNA protective activities of wine extracts from four indigenous Greek varieties by using a combination of customary tools. These standardized protocols include the readouts described in this study, which represent fundamental pillars for assessing the role of wine extracts in redox biology and, subsequently, their implication in diseases. Moreover, the evaluation of wine extracts’ antioxidant activity is useful in the generation of high-quality nutritional supplements with characterized bioactivity, and it serves as a baseline study for approaching the molecular mechanism of action of wine extracts to suggest potent target molecules for drug development in oxidative stress-related diseases.

DPPH^•^ and ABTS are synthetic free radicals commonly used for the quick estimation of antioxidant activity of hydrophilic and lipophilic compounds [59]. Another extensively used method is the reducing power assay that allows us to determine the ability of a phytochemical compound to donate electrons that reduce oxidized intermediates [60]. In contrast, OH^•^ and ^•^O_2_^−^ are endogenously generated radicals; thus, their estimation creates a prerequisite for a holistic approach to determine protection against both synthetic free radicals and endogenously generated free radicals (Figure 3) [61]. Moreover, we tested the protective efficacy of wine extracts against DNA strand breaks induced by peroxyl radical generation through “azo” initiators of peroxyl radicals. Additionally, we evaluated the effect of wine extracts against tert-induced mutagenicity using the Ames test.

As is shown in Figure 1 and Figure 2, red wine samples exhibited differentiated and elevated polyphenolic content; this is in line with the presence of tannins and anthocyanins in red wine varieties that has been described previously [62,63]. Among all four of our samples, Agiorgitiko stood out as the one that possessed the highest levels of phenolics, followed by the red variety, Xinomavro, the white variety, Malagouzia, and the white variety, Assyrtiko. Thus far, very few studies have examined the chemical composition of these emblematic Greek wine varieties, rendering a direct comparison of our findings with the existing literature challenging. Additionally, most of these studies did not include samples from all four varieties. However, in a comprehensive study published by Kallithraka et al., wines of the Agiorgitiko variety are also placed among the top samples in total phenol content [64]. Moreover, our findings showcased that Agiorgitiko tops other samples in phenolic acid and hydroxycinnamate levels, especially in relation to gallic acid, caftaric acid, coutaric acid, *p*-coumaric acid, syringic acid, and fertaric acid; this finding has been observed in previously published research on Greek red wine phenol composition [65]. In the case of flavonoids, most identified metabolites were either aglycones or glycosylated forms of quercetin, apigenin, luteolin, and kaempferol. Even though the red wine samples exhibited elevated flavonoid levels, such as rhamnetin and quercetin, the Malagouzia white variety had levels equal to those of Xinomavro. Furthermore, the Malagouzia variety was the only extract tested in which stilbene resveratrol was detected in traces; therefore, it was excluded from the inter-sample comparison. Nevertheless, we hereby have to note that previous studies on Greek wine samples have demonstrated that resveratrol in its free form is less abundant in local varieties compared to other wines, and its presence greatly depends on cultivation conditions and vinification processes [66,67,68,69,70,71,72]. Similar findings concerning phenolic compounds levels have also been reported in white wine varieties [73]. Finally, it should be noted that different bound forms of resveratrol, such as oligomers, were identified.

It has been reported that *p*-coumaric acid reduces the steatosis of liver cells and lipid aggregation in hepatic tissue in high-fat-diet mice [74]. This was the first study to connect this phenolic compound with hyperlipidemia, and subsequently with atherosclerosis. The Agiorgitiko variety was found to exert the most promising results regarding O_2_^−^ radical scavenging, mutagenicity (Ames test), and ROO^•^-induced DNA damage. Previous report on fertaric and caftaric acids indicate that their levels are elevated in the Agiorgitiko variety, which is mainly responsible for this variety’s antioxidant activity [75]. These results are also consistent with other studies in wines and grape stem extracts [76,77,78,79]. The other red wine variety, Xinomavro, exhibited almost similar IC_50_ values in ABTS and DPPH^•^ radical scavenging assays compared to Agiorgitiko, while it showed an increase in the reducing power assay; thus, the Xinomavro-derived extract is the most potent among all extracts tested in this respective assay. Surprisingly, the Assyrtiko white wine variety extract exerted the strongest OH^•^ radical scavenging ability among all extracts tested. All previous data indicate the heterogeneous presence of bioactive compounds between red and white wine extract varieties, directly affecting their antioxidant capacities. Additionally, in a previous study from our lab in which we used the same methodologies and experimental equipment, we described the antioxidant capacity of the known antioxidant ascorbic acid (vitamin C) [80]. A comparison between the results of these two studies revealed that vitamin C is more capable of scavenging DPPH^•^ (Vit C IC_50_; 4 ± 0.1μg/mL), ABTS (Vit C IC_50_; 3 ± 0.4μg/mL), and hydroxyl radical (Vit C IC_50_; 21 ± 0.1μg/mL) compared to all four of the wines tested. Concerning reducing power capacity, only the Xinomavro variety was equally potent compared to vitamin C (IC_50_; 5 ± 0.6 μg/mL), while all other wine extracts had a decreased capacity for protecting metals ions from reduction. Similarly, vitamin C (ΙC_50_; 300 ± 30.6 μg/mL) has a higher antigenotoxic activity than all wines tested. On the contrary, the antimutagenic capacity of vitamin C is lower (ΙC50; 164 ± 3.1 μg/mL) than that exerted by the wine extracts. Moreover, the synergetic effect of the phenolic compounds of each variety renders their antioxidant capacity dependent on the concentration of each compound, as well as on its in-between interaction and chemical structure [81]. However, their molecular mechanism of action remains elusive. Thus, further in cell-based and in vivo mechanistic analyses should be performed to investigate the action of these specific wine varieties.

Additionally, further studies are needed to elucidate the scavenging efficacy of each polyphenol, either by itself or synergistically, against these radicals; however, these studies are extremely laborious and costly. On the other hand, this study offered information concerning both polyphenol content and antioxidant capacity. Extracts derived from the Agiorgitiko variety were more abundant in terms of polyphenols, with enriched representation in the majority of the phenolic acids (e.g., syringic and ferulic acid), hydroxycinnamates (e.g., p-coumaric and caftaric acid), flavonoids (e.g., laricitrin and kaempferol), and flavonoid glucosides (e.g., apigenin-O-hexoside, quercetin-O-gluconide). At the same time, it was the extract with the highest capacity for scavenging radicals among almost all experimental approaches. The opposite correlation seems to be present in the extract of the Malagouzia variety, which has the lowest representation of polyphenols and least antioxidant capacity among the varieties tested. The above observations support the idea that polyphenol abundancy and polyphenol synergy can boost the antioxidant activity of edible goods. Moreover, although moderate wine consumption has been associated with chemoprevention in several diseases, the global literature lacks a solid methodology array to evaluate the bioactivity of wine extracts for determining a well-characterized set of readouts. The proposed methodology can estimate the radical scavenging activity of synthetic radicals, take into consideration both lipophilic and hydrophilic molecules, assess biologically important endogenous free radicals, and determine the alleviating effect on macromolecule damage. Furthermore, this methodology allows for comparison with future studies, thus demonstrating its repeatability and scientific integrity.

Based on our results, all tested wine extracts seemed to exhibit potent antioxidant, antimutagenic and antigenotoxic activities. Although further cell-based and in vivo studies are required, the wine extracts possess a high bioactivity that can make an impact in the food additive and medicinal product industries. More large studies examining different batches and crops of these wine varieties should also be performed to develop insights that would fully characterize their biofunctional properties. In conclusion, a significant proportion of the antioxidant activity of wine may be attributed to compounds such as p-coumaric acid and caftaric acid, but scarce information is available regarding the extent of these compounds’ absorption and bioavailability. Finally, a correlation between high bioavailability and high antioxidant activity has not been established, and further studies including pharmacokinetics should be performed to assess phenolic compounds’ nutritional benefits and their action on redox-related diseases.

## 4. Materials and Methods

### 4.1. Chemicals and Reagents

LC–MS grade solvents were purchased from Merck Chemicals (Darmstadt, Germany) and high purity water was provided by a Millipore Direct-Q^®^ 3 UV purification system (MilliporeSigma, Burlington, MA, USA). Pierce™ LC–MS grade formic acid was obtained from Thermo Fisher Scientific (Waltham, MA, USA). Gallic acid, Folin–Ciocalteu reagent and sodium carbonate were purchased from Sigma-Aldrich (St. Louis, MO, USA).

### 4.2. Sample Preparation 

Wines from the four Greek flagship varieties, two from white (Asyrtiko, Malagouzia) and two from red (Xinomavro and Agiorgitiko) grapes, were selected. Vinification process included the fermentation and filtration of generated wine in inox vessels without further maturation in wooden barrels. All wine varieties tested were produced in the year 2020. A basic enological analysis of these wine varieties is presented in Table S2. To remove the contained ethanol, all wines were concentrated in a rotary evaporator (Büchi Labortechnik AG, Flawil, Switzerland) at 40 °C until their volume was reduced by half. Subsequently, an absorption resin treatment was employed using polymeric resin (Amberlite^®^ XAD-4, Supelco, Bellefonte, PA, USA), and the wines were consecutively recovered using analytical grade isopropanol (Fischer Scientific, Pittsburg, PA, USA). Samples were once again dried in a rotary evaporator and lyophilized for complete solvent removal, weighted, and stored in −20 °C. Finally, all 4 varieties were sampled using similar protocols simultaneously to allow us to compare their relative differences in phenol content and antioxidant efficacy.

### 4.3. UHPLC-ESI-TripleTOF-HRMS Analysis

Liquid chromatography analysis was performed on an ΕxionLC™ system (AB Sciex, Framingham, MA, USA). Detection was performed on a Sciex TripleTOF^®^ 5600+ mass spectrometer equipped with a DuoSpray™ ion source operated in the negative ESI mode, both for calibration and data acquisition. Calibration was performed using a calibrant delivery system (CDS) (AB Sciex). A total of 10 μL of each extract at 250 μg/mL was injected into the system. Separation was achieved on a Fortis Speedcore^®^ (Fortis Technologies Ltd., Cheshire, UK) C18 column (10 cm × 2.1 mm, 2.6 μm) using a water gradient containing 0.1% (*v/v*) formic acid (A) and acetonitrile (B). Elution started at 5% B, which was maintained for 2 min and increased to 100% B in another 13 min. These conditions were kept for 2 min before returning to initial conditions for 1 min for a 4-min re-equilibration (22 min in total). Column temperature was maintained at 40 °C and the flow rate was set to 0.4 mL/min. The mass spectrometer was operated in the information-dependent acquisition (IDA) mode using a TOF-MS survey scan of 100–1000 Da and 1 dependent TOFMS/MS scan of 50–1000 Da, while accumulation time was set to 0.25 and 0.07 sec for each experiment, respectively. Declustering potential was set to 80, while collision energy (CE) was set to −40 V, with a spread of ±15 V. For the ESI source, temperature was set to 450 °C and ion spray voltage was −4500 V. Source gas and exhaust gas were set to 50 psi, while curtain gas was set to 35 psi. Data acquisition was performed using Analyst^®^ 1.7.1 software (AB Sciex) and spectral interpretation was performed using the Sciex OS software platform (AB Sciex). Peak areas for the detected metabolites were calculated using the “suspect screening” streamline. Each generated entry was manually checked to remove inauthentic features, and compound identification was based on mass score, RDB equivalents, isotopic ratio matching, formula finder score, and HRMS/MS data.

### 4.4. In Vitro Biomarkers for the Assessment of the Wine Extracts’ Antioxidant Activity

Samples were tested for their polyphenolic content and their antioxidant efficacy using the following experimental protocols. For all experimental methodologies that were performed in this study, we conducted at least 2 independent experiments including 3 technical replicates, apart from the plasmid methodology in which we ran 2 independent experiments using 1 technical replicate due to the nature of the experimental protocol.

#### 4.4.1. Determination of Total Polyphenolic Content (TPC)

The total polyphenolic content (TPC) of 4 wine extracts was determined using the Folin-Ciocalteu reagent, as previously described [82]. More specifically, a total of 20 µL of each extract (2 mg/mL) was added to a tube containing 1 mL of distilled water, followed by the addition of 100 µL of Folin-Ciocalteu reagent and incubation for 3 min at room temperature. Subsequently, 280 µL of 25% *w/v* sodium carbonate solution, along with 600 µL of distilled water, was added to the mixture. Finally, following 1 h incubation at room temperature in the dark, absorbance was determined at 765 nm. The measurement was carried out on a Hitachi U-1900 radio beam spectrophotometer (serial no. 2023-029; Hitachi, Ltd., Tokyo, Japan). The optical density of the sample without the Folin–Ciocalteu reagent at 765 nm was also measured and subtracted from the respective measurement that the complete mixture exhibited. The TPC was determined using a gallic acid standard curve (50–1500 µg/mL). The TPC was presented as µg of gallic acid equivalents per mg of extract.

#### 4.4.2. Determination of DPPH^•^ Radical Scavenging Activity

The free-radical scavenging activity (RSC) of wine extracts was evaluated by a 2,2-Diphenyl-1-picrylhydrazyl (DPPH^•^) radical assay [83], as previously described, with slight modifications [49,84,85,86]. Briefly, 900 μL of methanol and 50 μL of freshly prepared methanolic solution of DPPH^•^ radical (2 mM) were mixed with 50 μL of each wine extract in different concentrations (ranging from 1.56 to 150 μg/mL). The contents were vigorously mixed, incubated at room temperature in the dark for 20 min, and the absorbance was recorded at 517 nm. The measurement was conducted on a Hitachi U-1900 ratio beam spectrophotometer (Hitachi). In each analysis, 1 mL of methanol was used as blank and 50 μL of DPPH^•^ along with 950 μL methanol, was used as assay control. The final reaction volume of all samples was 1 mL.

The percentage RSC of the tested extracts was calculated using the following equation:

(1) $$\%\;DPPH\;radical\;scavenging\;activity\;=\frac{Abs_{control}-Abs_{sample}}{Abs_{control}}\times 100$$

where *Abs_control_* and *Abs_sample_* were the values of absorbance from control and tested wine extracts, respectively. Moreover, the IC_50_ value, defined as the concentration of sample leds to a 50% decrease in the DPPH^•^ radical, was used to compare the radical scavenging efficiency of the extracts. All analyses on tested samples were carried out in triplicate and at least two experiments were conducted.

#### 4.4.3. Determination of ABTS Radical Scavenging Activity

The radical scavenging activity of 2,2′-Azinobis-(3-ethylbenzothiazoline-6-sulfonic acid) (ABTS^+•^) [87] was measured, with minor modifications, as described previously [49,88]. In brief, ABTS^+•^ radicals were produced by mixing 500 μL of ABTS (1 mM) with 50 μL of H_2_O_2_ (30 μM) and 50 μL of horseradish peroxidase (HRP) (6 μM) (Sigma-Aldrich) in 430 μL of dH_2_O. Immediately following the addition of HRP, the contents were vigorously mixed, incubated at room temperature in the dark for 45 min, and the reaction was monitored at 730 nm until optical density was stabilized. Subsequently, 50 μL of each wine extract, in a concentration range from 0.78 to 100 μg/mL, was added to the reaction mixture, and absorbance was determined at 730 nm. In each analysis, one sample with 500 μL of ABTS^+•^ (1 mM) and 50 μL of H_2_O_2_ (30 μM) in 450 μL dH_2_O was used as a blank, while one sample with 500 μL of ABTS^+•^ (1 mM), 50 μL of H_2_O_2_ (30 μM), and 50 μL of horseradish peroxidase (HRP) (6 μM) in 400 μL dH_2_O was used as a control. Each tested sample had its own control with the same reagent volumes, except HRP. The final reaction volume was 1050 μL. All analyses were carried out in triplicate and at least two experiments were conducted. The ABTS^+•^ radical scavenging activity was calculated according to the equation:

(2) $$\%ABTS^{+•}radical\;scavenging\;activity\;=\frac{Abs_{control}-Abs_{sample}}{Abs_{control}}\times 100$$

where *Abs_control_* and *Abs_sample_* were the values of absorbance acquired from control and tested wine samples, respectively. Moreover, the IC_50_ value, defined as the concentration of the sample that led to a 50% decrease in the ABTS^+•^ radical, was used to compare the radical scavenging efficiency of extracts. All analyses on tested samples were carried out in triplicate and at least two experiments were conducted.

#### 4.4.4. Determination of the Reducing Power Assay

The reducing power assay was adapted from Yen et al. [60] and determined with slight modifications [89,90]. Specifically, 50 μL of each wine extract at different concentrations (ranged from 0.78 to 100 μg/mL) was dissolved in 200 μL of phosphate buffer (0.2  M, pH 6.6), mixed with 250 μL of 1% potassium ferricyanide, and incubated at 50 °C for 20  min. Subsequently, samples were placed on ice for 5  min and 250  μL of 10% TCA was added, followed by a centrifugation (3000 rpm, 10  min at 25 °C). After centrifugation, supernatant was transferred into a new tube, and 250  μL of dH_2_O and 50  μL of 0.1% ferric chloride were added, followed by a 10 min incubation at room temperature in the dark. Absorbance was determined at 700  nm.

(3) $$AU_{0.5}=\frac{Abs_{control}-Abs_{sample}}{Abs_{control}}$$

where *Abs_control_* and *Abs_sample_* were the values of absorbance from control and tested wine samples, respectively. AU _0.5_ value was defined as the sample concentration with an absorbance value at 0.5 at 700  nm, and absorbance values of different sample concentrations were plotted and calculated on a graph. All analyses were carried out in triplicate and at least two experiments were conducted.

#### 4.4.5. Determination of Superoxide Radical (O_2_**^•^**^−^) Scavenging Activity

Superoxide (O_2_^•−^) radical scavenging activity was determined using a slightly modified protocol from Gülçin et al. [91]. According to this protocol, superoxide anion (O_2_^•−^) was generated in a phenazine methosulfate and reduced nicotinamide adenine dinucleotide (PMS-NADH) system through NADH oxidation, and it reduced nitroblue tetrazolium (NBT^2+^; yellow) to formazan (blue) [92]. Briefly, in 625 μL of Tris-HCl (16 mM, pH 8.0), 125 μL of NBT (300 μM), 125 μL of NADH (468 μM) and 50 μL of each wine extract (ranging from 3.1 to 400 μg/mL) were added at different concentrations. The reaction was started by adding 125 μL of PMS (60 μM). The samples were incubated for 5 min and absorbance was measured at 560 nm. In each experiment, the samples without PMS were used as blanks and the samples without wine extracts were used as controls. The superoxide (O_2_^•−^) radical scavenging activity was calculated according to the equation:

(4) $$\%\mathrm{Superoxide}\;\mathrm{radical}\;\mathrm{scavenging}\;\mathrm{activity}\;=\frac{Abs_{control}-Abs_{sample}}{Abs_{control}}\times 100$$

where *Abs_control_* and *Abs_sample_* were the values of absorbance from control and tested wine extract samples, respectively. Moreover, the percentage inhibition and the IC_50_ value, defined as the concentration of the sample that led to a 50% decrease in the O_2_^•−^ radical, were used to compare radical scavenging efficiency among extracts. All analyses on tested samples were carried out in triplicate and at least two experiments were conducted.

#### 4.4.6. Determination of Hydroxyl Radical (OH^•^) Scavenging Activity

Hydroxyl (OH^•^) radical scavenging activity was determined using a method from Chung et al. [93], with slight modifications, as previously described [49,94]. Specifically, 50 μL wine extracts at different concentrations (ranged from 25 to 800 μg/mL) were added to 225 μL of sodium phosphate buffer (0.2 M, pH 7.4), 75 μL of 2-deoxyribose (5 mM), 75 μL of FeSO_4_-EDTA (10 mM), 250 μL of H2O, and 75 μL of H_2_O_2_ (10 mM), and the samples were incubated at 37 °C for 1 h. After incubation, 375 μL of TCA (2.8%) and και 375 μL of 2-thiobarbituric acid (1% dissolved in 50 mM NaOH) were added and samples were incubated at 95 °C for 10 min. Then, the samples were placed on ice for 5 min and centrifuged at 3000 rpm for 10 min at 25 °C. Absorbance was measured at 520 nm. In each experiment, one sample without H_2_O_2_ was used as blank and samples without wine extracts were used as control. All analyses were carried out in triplicate and at least two experiments were conducted. The OH^•^ radical scavenging activity was calculated according to the equation:

(5) $$\%\;{\mathrm{OH}}^{•}\mathrm{radical}\;\mathrm{scavenging}\;\mathrm{activity}=\frac{Abs_{control}-Abs_{sample}}{Abs_{control}}\times 100$$

where *Abs_control_* and *Abs_sample_* were the values of absorbance from control and tested wine samples, respectively. The IC_50_ value, defined as the concentration of the sample that led to a 50% decrease in the OH^•^ radical, was used to compare the radical scavenging efficiency of extracts. All analyses on tested samples were carried out in triplicate and at least two experiments were conducted.

#### 4.4.7. Determination of Peroxyl Radical-Induced DNA Plasmid Strand Cleavage

The assay of DNA relaxation from peroxyl radicals has already been described [49,85,94,95,96]. The plasmid (pBluescript SK+, Fermentas, Waltham, MA, USA) DNA normally exists in the supercoiled conformation but, following a single-strand break, it is converted to an open circular conformation. This formation is an indication of oxidative modification. Based on this principle, the protective activity of each wine extract against DNA single-strand breaks by AAPH (2.5  mM) was assessed. Briefly, in a total reaction volume of 10  μL, 2  μL (4 μg/mL) of plasmid DNA was mixed with PBS and a range of different concentrations of the tested wine extracts. Specifically, the tested concentrations ranged from 6.25 to 800 μg/mL and from 250 to 2000 μg/mL for red and white wines extracts, respectively. The tubes were incubated for 45  min at 37 °C. Finally, 3  μL of loading buffer (containing bromophenol blue 0.25%  +  30% glycerol) was mixed to terminate the reaction, and the samples were loaded on a 0.8% agarose gel. The samples were run at 70 V for 60  min. Subsequently, 12.5 μL of ethidium bromide (10 mg/mL) in 250 mL of dH_2_O was used to stain the gel for 30  min. Consequently, the agarose gel was washed with 250  mL of dH_2_O for 30  min. Finally, the agarose gel was exposed to UV, the MultiImage Light Cabinet (Alpha Innotech, San Leandro, CA, USA) for image acquisition, and the results were analyzed with Alpha View software. For negative control, DNA was mixed with PBS only, while the positive control was defined as the plasmid DNA that was introduced in the PBS and AAPH mixture. The highest extract concentration tested was also mixed with DNA and PBS, without the AAPH, to ascertain the putative effects of the extracts on plasmid DNA. None of these tested concentrations induced DNA breaks. The percentage inhibition was calculated using the following equation:

(6) $$\%\;Inhibition=\frac{S-S_{o}}{S-S_{control}}\times 100$$

where *S_control_* is the percentage of the supercoiled DNA of the negative control sample (plasmid DNA alone), *S* is the percentage of the supercoiled plasmid DNA of the positive control sample (without the tested extracts but in the presence of the radical initiating factor), while *So* is the percentage of the supercoiled plasmid DNA of the sample with the tested wine extracts and the radical initiating factor. The IC_50_ value that was defined as the concentration of the sample that led to a 50% inhibition of the AAPH radical was used to compare the protective activity of each wine extract against DNA single-strand breaks by this radical. All analyses on tested samples were carried out in triplicate and at least two experiments were conducted.

#### 4.4.8. Determination of Antimutagenic Capacity Using an Ames Test

The antimutagenic capacity of the tested wine extracts was evaluated through the application of an Ames test using Salmonella *typhimurium* bacterium strain TA102 (MolTox), as reported by Maron and Ames and previously described [85,97]. Briefly, 700 μL of the bacterium culture was used to inoculate 30 mL of autoclaved Oxoid nutrient broth no. 2. Cultures were placed on a vibrator (100 rpm) and incubated in the dark at 37 °C until the cells reached a density of 1–2 × 10^9^ colony forming units (CFU/mL, OD540 between 0.1 and 0.2). Plates with oxidant and 50 μL of each wine extract at various concentrations, 2 mL of top agar, 100 μL of the bacterial culture, 50 μL of tert-butyl hydroperoxide (0.4 mM) were added in sterile tubes. More specifically, tested concentrations of red wines extracts ranged from 2.5 to 40 μg per extract/plate, while white wines ranged from 2.5 to 100 μg per extract/plate. Additionally, a plate with the oxidizing agent alone and a plate without the oxidizing agent or the tested extract were used as positive and negative control, respectively.

The two highest concentrations of every wine extract were also assayed alone to determine putative induction of mutations. An incubation at 37 ± 2 °C for 48 h was followed in all the generated sample tubes that were poured onto plates covered by glucose minimal agar; the histidine revertant colonies (His+) were subsequently counted. The number of induced revertants was obtained by subtracting the number of spontaneous revertants from the number of revertants on the plates with the mutagen and/or antioxidant.

The percentage inhibition of mutagenicity was calculated as follows:

*% Inhibition* = No. of colonies per plate with oxidant + tested extract number of colonies per plate with oxidant alone × 100. (7)

### 4.5. Statistical Analysis

Data were analyzed using one-way ANOVA followed by Dunnett’s tests for multiple pairwise comparisons, using the statistical package SPSS (version 21.0 SPSS Inc., Chicago, IL, USA). All data were presented as mean ± SD (i.e., standard deviation) and differences of *p* < 0.05 were considered statistically significant.

## Figures and Tables

**Table 1:** List of compounds included in the inter-sample comparison.

Component Name	Retention Time (min)	Pseudomolecular Ion [M-H]^−^	Mass Error (ppm)	Molecular Formula
gallic acid	1.02	169.014	1.053	C_7_H_5_O_5_
protocatechuic acid	1.93	153.019	0.749	C_7_H_5_O_4_
hydroxytyrosol	2.3	153.056	0.968	C_8_H_9_O_3_
caftaric acid	2.86.	311.041	0.848	C_13_H_11_O_9_
gentisic acid	3.49	153.019	0.526	C_7_H_5_O_4_
coutaric acid	4.39	295.046	0.998	C_13_H_11_O_8_
*p*-coumaric acid	4.39	163.04	−0.144	C_9_H_7_O_3_
caffeic acid	4.76	179.035	1.149	C_9_H_7_O_4_
fertaric acid	4.82	325.057	2.367	C_14_H_13_O_9_
ferulic acid	4.83	193.051	2.154	C_10_H_9_O_4_
tyrosol	5.45	137.061	1.194	C_8_H_9_O_2_
syringic acid	5.5	197.046	2.384	C_9_H_9_O_5_
rutin	5.69	609.146	0.796	C_27_H_29_O_16_
ellagic acid	5.71	300.999	1.724	C_14_H_5_O_8_
quercetin-*O*-hexoside	5.76	463.088	0.819	C_21_H_19_O_12_
quercetin-*O*-glucuronide	5.79	477.067	0.706	C_21_H_17_O_13_
kaempferol-*O*-hexoside	5.84	447.093	0.270	C_21_H_19_O_11_
piceid	6.25	389.124	−0.036	C_20_H_21_O_8_
taxifolin	6.45	303.051	0.203	C_15_H_11_O_7_
apigenin-*O*-hexoside	6.5	431.098	1.841	C_21_H_19_O_10_
astringin	6.59	405.119	0.785	C_20_H_21_O_9_
chlorogenic acid	6.65	353.088	1.944	C_16_H_17_O_9_
luteolin	7.01	285.04	3.032	C_15_H_9_O_6_
quercetin	7.03	301.035	2.187	C_15_H_9_O_7_
apigenin	7.56	269.046	0.020	C_15_H_9_O_5_
kaempferol	7.66	285.04	1.626	C_15_H_9_O_6_
hesperetin	7.78	301.072	1.188	C_16_H_13_O_6_
laricitrin	7.81	331.046	0.803	C_16_H_11_O_8_
rhamnetin	7.81	315.051	0.242	C_16_H_11_O_7_

**Table 2:** Total Polyphenolic Content (TPC) of different wine varieties.

	Sample Name	TPC (mg GA/g Extract)
Red wines	Xinomavro	267.1
	Agiorgitiko	265.4
White wines	Assyrtiko	155.7
	Malagouzia	81.1

**Table 3:** Antigenotoxic activity of the tested wine extracts using a plasmid relaxation assay.

Plasmid Relaxation Assay			
Red Wines IC_50_ (μg/mL)		White Wines IC_20_ (μg/mL)	
Xinomavro	Agiorgitiko	Assyrtiko	Malagouzia
260.5 ± 27.4 ^a^	116.1 ± 19.4 ^b^	220.3 ± 14.1	150.1 ± 15.0

Results are expressed as mean ± SD. ^a, b^: means without a common letter depict significant difference (*p* < 0.05).

**Table 4:** Antimutagenic capacity of the tested wine extracts using Ames test.

Ames Test		
	Sample Name	IC_50_ (μg/mL)
**Red wines**	Xinomavro	8.0 ± 0.02 ^a^
	Agiorgitiko	7.5 ± 0.37 ^a^
**White wines**	Assyrtiko	16.9 ± 0.24 ^b^
	Malagouzia	25.9 ± 0.30 ^c^

Results are expressed as mean ± SD. ^a–c^: means without a common letter are statistically significantly different (*p* < 0.05).

## Data Availability

All data are available upon request from the corresponding author.

## Supplementary Materials

The following are available online at https://www.mdpi.com/article/10.3390/metabo11070436/s1. Supplementary Tables S1 and S2 are available online along with the manuscript. Table S1. Peak area comparison of the selected features among the four Greek wine varieties. Table S2. Basic enological analysis for all wine varieties tested.
