# Supplementary material for: Assessment of Antioxidant and Antimutagenic Properties of Red and White Wine Extracts In Vitro
Source: Metabolites. 2021 Jul 2;11(7):436. doi: 10.3390/metabo11070436 (PMC8304343; doi:10.3390/metabo11070436)
Supplement: Supplementary file 1 [file metabolites-11-00436-s001.zip › metabolites-1244048-supplementary.pdf]

Table S1. Peak area comparison of the selected features among the four Greek wine varieties.

Component Name	Peak Area			
	Asyrtiko	Malagouzia	Agiorgitiko	Xinomavro
gallic acid	6.02E + 06	4.11E + 06	9.23E + 06	3.80E + 05
protocatechuic acid	4.60E + 05	2.52E + 05	8.40E + 05	4.48E + 05
hydroxytyrosol	4.39E + 05	1.36E + 05	4.75E + 05	8.86E + 04
caftaric acid	5.98E + 06	8.99E + 06	6.01E + 07	3.44E + 07
gentisic acid	7.25E + 04	7.81E + 04	2.24E + 05	3.64E + 05
coutaric acid	3.56E + 06	5.03E + 06	2.57E + 07	8.81E + 06
p-coumaric acid	2.79E + 05	3.75E + 05	1.81E + 06	4.49E + 05
caffeic acid	3.96E + 05	7.86E + 05	2.72E + 06	5.11E + 06
fertaric acid	1.09E + 06	7.90E + 05	1.46E + 07	7.77E + 06
ferulic acid	6.48E + 04	3.74E + 04	6.67E + 05	3.66E + 05
tyrosol	1.09E + 03	2.95E + 03	6.35E + 04	1.52E + 05
syringic acid	2.13E + 06	1.78E + 06	3.89E + 06	1.50E + 05
rutin	2.28E + 04	2.45E + 04	4.43E + 05	2.71E + 04
ellagic acid	4.20E + 05	8.71E + 05	3.71E + 05	5.31E + 05
quercetin-*O*-hexoside	7.34E + 04	6.65E + 04	1.62E + 06	7.22E + 04
quercetin-*O*-glucuronide	1.75E + 06	1.25E + 06	4.74E + 06	1.42E + 05
kaempferol-*O*-hexoside	1.46E + 05	5.81E + 04	5.85E + 05	5.95E + 04
piceid	8.71E + 04	4.83E + 05	1.55E + 05	4.70E + 04
taxifolin	1.82E + 04	1.36E + 04	9.11E + 04	4.64E + 04
apigenin-*O*-hexoside	2.65E + 04	4.76E + 04	1.19E + 06	5.16E + 05
astringin	3.95E + 04	1.53E + 04	3.18E + 04	8.75E + 03
chlorogenic acid	3.23E + 04	2.33E + 04	1.82E + 06	7.13E + 05
luteolin	8.61E + 04	7.49E + 04	2.89E + 05	3.56E + 05
quercetin	1.31E + 05	3.86E + 05	4.28E + 05	2.93E + 05
apigenin	4.81E + 03	6.35E + 03	9.78E + 03	1.30E + 04
kaempferol	3.13E + 03	6.73E + 03	4.50E + 05	1.45E + 05
hesperetin	3.50E + 03	4.02E + 03	2.39E + 04	2.05E + 04
laricitrin	8.64E + 02	2.73E + 02	1.19E + 05	1.40E + 03
rhamnetin	3.32E + 04	7.56E + 04	6.29E + 04	1.51E + 04

Table S2. Basic enological analysis for all wine varieties tested.

	Total acidity	pH	Vol (%)	Volatile acidity	Phenol Index (280)	A420	E	A	Sulfurous anhydride	
									Free	Total
**Malagouzia**	5.4	2.95	12.44	0.27	6.6	0.073	-	-	23	77
**Asyrtiko**	6.2	2.94	12	0.36	11.5	0.137	-	-	30	92
**Agiorgitiko**	5.9	3.45	12.95	0.54	41.5	-	6.9	0.75	14	90
**Xinomavro**	6.1	3.35	12.15	0.48	47.9	-	6.8	0.76	11	94
